# A reassessment of trends and rural–urban/regional differences in the total fertility rate in China, 2000–2020: analyses of the 2020 national census data

**DOI:** 10.1038/s41598-024-59177-2

**Published:** 2024-04-13

**Authors:** Long Li, Guangzhao Jin, Xiaozhen Lai, Rize Jing, He Zhu

**Affiliations:** 1 Center for Population and Development Studies, Renmin University of China, Beijing, 100872 China; 2 China Center for Health Development Studies, Peking University, Beijing, 100871 China; 3 School of Public Administration and Policy, Renmin University of China, No. 59, Zhongguancun Street, Haidian District, Beijing, 100872 China; 4 China Center for Health Development Studies, Peking University, No. 38, Xueyuan Road, Haidian District, Beijing, 100191 China

**Keywords:** Health care, Risk factors

## Abstract

The decline in the total fertility rate (TFR) is a key driver of population change and has important implications for population health and social development. However, China’s TFR has been a considerable controversy due to a lack of high-quality data. Therefore, this study used the 2020 national population census of China (NPCC) data and reverse survival method to reassess temporal trends in the TFRs and to reexamine rural–urban differences and regional variations in TFRs from 2000 to 2020 in China. Overall, there were significant gaps between the estimated and reported TFRs before 2020, and the estimated TFRs based on the 2020 NPCC data remained higher than the reported TFRs from government statistics. Although TFRs rebounded shortly in the years after the two-child policy, they have shown a wavelike decline since 2010. Additionally, the estimated TFRs fluctuated below 1.5 children per woman in urban areas compared to above 1.8 in rural areas, but the rural–urban differences continued to decrease. Regarding geographic regional variations, the estimated TFRs in all regions displayed a declining trend during 2010–2020, especially in rural areas. Large decreases of over 25% in TFRs occurred in the north, east, central, and northwest regions. In addition to changing the birth policy, the government and society should adopt comprehensive strategies, including reducing the costs of marriage, childbearing, and child education, as well as promoting work-family balance, to encourage and increase fertility levels.

## Introduction

The total fertility rate (TFR), known as an estimate of lifetime fertility, is one of the most popular fertility rate measurements. The decline in the TFR is often attributed to reduced desires for children^1,2^, and it can shrink working-age share, exacerbating the burden of the pay-as-you-go systems and undermining sustainable development^3,4^.

The TFR is crucial to assess demographic changes and prepare for further development. However, the fertility levels in China, where the government performed a long period of strict birth control policy, have been a considerable controversy since the 1990s, as many demographers argued them inaccurate due to underreporting issues in the official statistical system^5^. According to the national population censuses, China’s TFR was 1.22 children per woman in 2000 and slightly decreased to 1.18 in 2010^6^, both of which were among the lowest national TFRs in the world at that time^7^. Unfortunately, they were misunderstood and widely cited by non-professionals. Meanwhile, other estimated TFRs for two years from previous studies ranged from 1.4 to 1.8^8^. The potentially underestimated TFRs may be due to data collection difficulties in the context of massive rural-to-urban migration and avoidance of penalties under the one-vote-down family planning cadre responsibility system (i.e., failure to perform adequately in the area of fertility can lead to a major reduction in wages or even dismissal from one’s job)^9^. Previous studies have adopted various techniques to improve the estimation of fertility levels, yet few of them have reached an agreement because of the lack of high-quality raw data^10^. Thus, China’s TFRs over the past decades remain a puzzle. Despite considerable debates on fertility levels, China has begun to change its traditional birth control policy to address the predicted low TFRs recently, and a selective two-child policy (allowing couples with one spouse being an only child to have two children), a universal two-child policy, and a three-child policy were launched sequentially in 2013, 2015, and 2021, respectively^11,12^.

The TFR and its pace of decline varied across rural–urban areas and geographic regions given different sociodemographic and socioeconomic conditions. In China, the implementation of the strict birth control policy has manifested large regional differences since its beginning, especially between rural and urban areas^13^. The one-child policy was relatively strict to be enforced among urban residents, but rural families in most provinces were permitted to have a second child if the first was female due to certain feasibility concerns. Even in several provinces, mainly located in the southwest and northwest regions, all rural families were allowed to have a second child. Analyzing regional heterogeneities in fertility levels and identifying areas with very low TFRs are essential for designing targeted programs on better access to sexual and reproductive as well as maternal and newborn health services.

Inaccuracies in fertility estimates may hinder the understanding of fertility transition and health- and development-related outcomes. Most importantly, the improvements in data collection and quality in the 2020 national population census data provide the opportunity to examine the temporal trends in TFRs in China. Taken together, this study aims to use the newest 2020 national population census data (1) to assess temporal trends in TFRs in China from 2000 to 2020 to make a comparison with the reported TFRs from government statistics and (2) to examine the rural–urban differences and regional variations in TFRs between 2000 and 2020.

## Methods

### Data sources

#### The 2020 national population census of China

Population data were retrieved from the 2020 national population census of China (NPCC), conducted by the National Bureau of Statistics (NBS) of China from November 1 to December 10, 2020. Compared to previous NPCCs, the 2020 NPCC has made significant improvements in the data collection process to reduce underreporting and increase data accuracy^14^.

**Figure 1:**
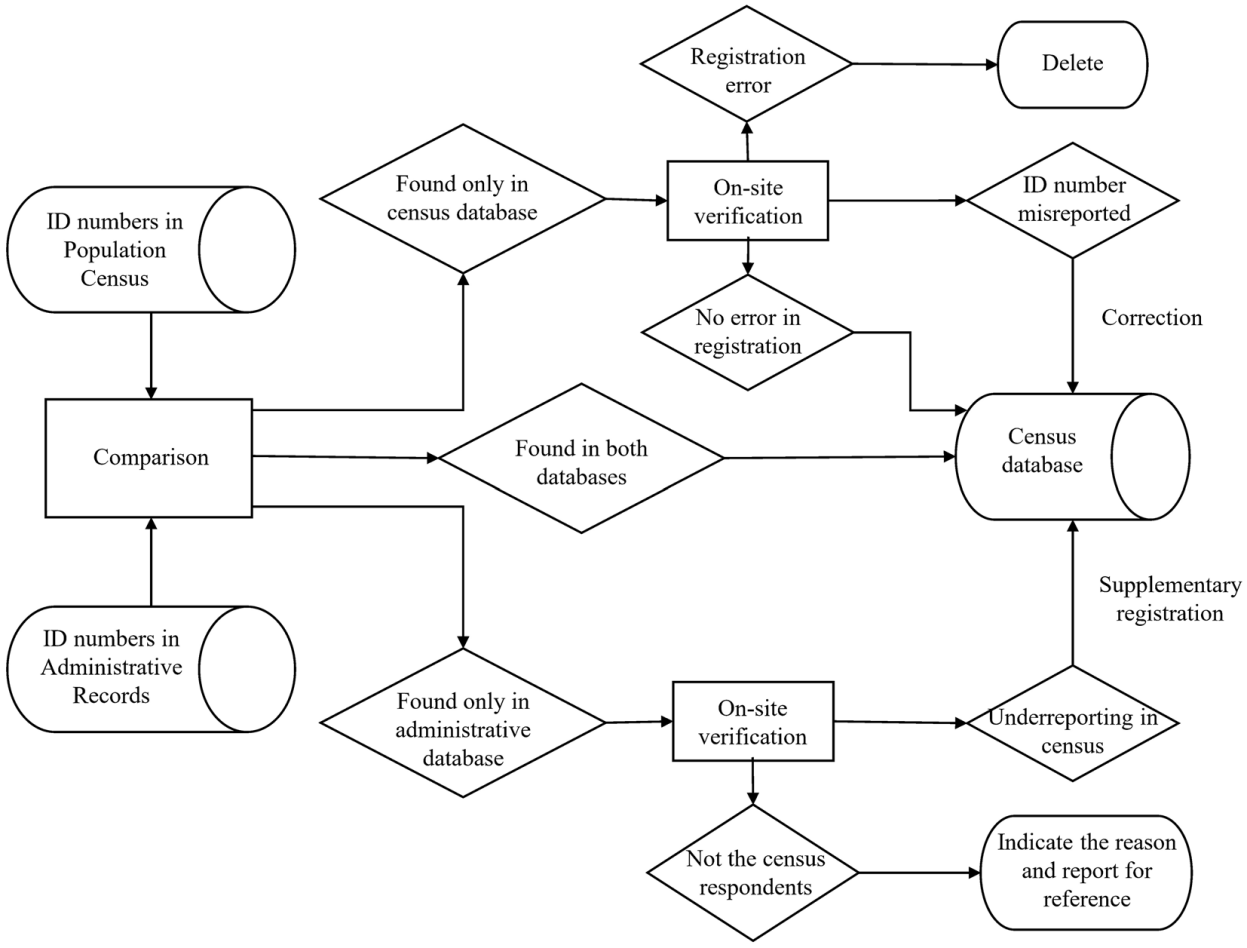
Process of cross-checking based on ID numbers between census data and administrative records in the 2020 national population census of China.

The 2020 NPCC was the first Chinese census to collect individual ID numbers, the unique and invariable legal number for each Chinese citizen. The government took the initiative to verify ID numbers in the census by using administrative records (e.g., household registration data and birth certificate data), which allowed it to capture the migrant population and identify potential missing misreported individuals in the census (Fig. 1). Moreover, the enhanced management systems of community residents during the COVID-19 pandemic helped reduce barriers to collecting data on the migrant population to improve coverage. Additionally, the 2020 NPCC fully employed information technologies in data collection including collecting data via electronic equipment, establishing an online registration channel, implementing online centralized management, and applying the big data of enterprises (more details refer to Appendix S1)^14^.

**Figure 2:**
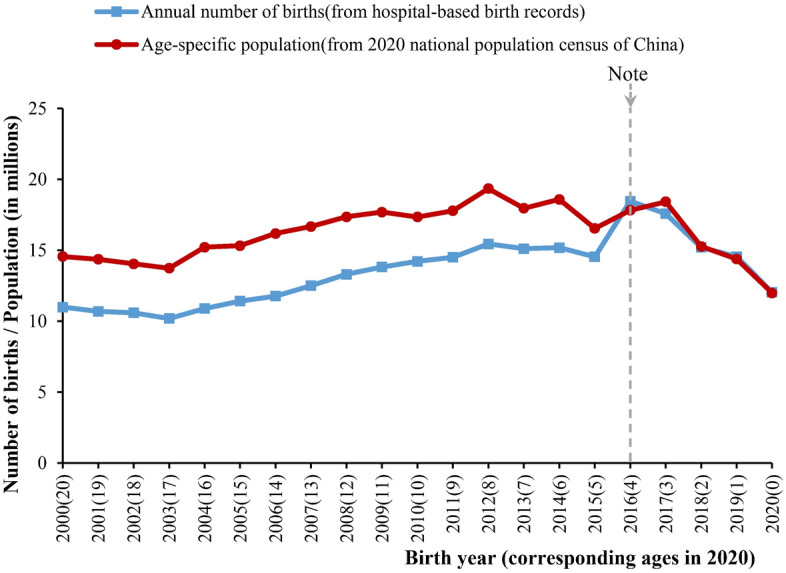
The number of births from the hospital-based birth records and the population of corresponding ages in the 2020 national population census of China. Note that before 2016, hospital-based birth records only recorded the live births of women with registered residences (a household registration system established in the 1950s in China). Since 2016, the live births of women without registered residence have also been recorded to lead to higher hospital delivery rates, and therefore it could largely reflect the total births in China.

According to the post-enumeration check results, the 2020 NPCC was recognized to have the highest quality in China’s population census history with the lowest underreporting rate of only 0.05% (Table 1). Some data independent from the NBS system could also prove the high quality of the 2020 NPCC. For instance, the numbers of births reported in hospital-based birth records published by the National Health Commission of China were close to those in the 2020 NPCC by corresponding ages (Fig. 2).

**Table 1:** Underreporting rates of national population censuses in China.

National population census of China	Underreporting rate (%)
The 1982 national population census	0.06
The 1990 national population census	0.07
The 2000 national population census	1.81
The 2010 national population census	0.12
The 2020 national population census	0.05

Data were extracted from major data communiques of the national population censuses released by the National Bureau of Statistics (NBS) of China.

#### Censuses and intercensal sample surveys from 2000 to 2019 in China

Except for the 2020 NPCC, this study also utilized censuses and intercensal sample surveys from 2000 to 2019 conducted by the NBS of China. Since 2000, the NBS of China has conducted three national censuses in 2000, 2010, and 2020, two 1% sample surveys in 2005 and 2015, and annual 1‰ sample surveys on population changes from 2001 to 2019 (excluding the years mentioned above). The previous censuses and sample surveys provided structural data as a reference, and this study did not use scaled data from previous censuses and sample surveys while reassessing fertility levels.

The censuses and sample surveys were all carried out through household interviews by trained enumerators. The questionnaire items were similar across all censuses or sample surveys. In the census, survey forms were divided into short form and long form. The short form covered sex, date of birth, ethnicity, residency, education status, etc., and was answered by all individuals who had Chinese nationality and resided in China. The long form covered all the items in short form and included more detailed items regarding marriage, childbirth during last year, occupation, etc., and was answered by a 10 percent sample of randomly selected households. For the 1%/1‰ sample survey, the long form was taken to collect the information of households selected through stratified, multistage, and proportional probability sampling procedures.

### Study variables

#### Total fertility rates (TFRs)

We used TFRs to measure the fertility levels. The TFR is defined as the average number of children that a cohort of women would have at the end of their reproductive period if they were subject during their reproductive lives (aged 15–49 years) to the age-specific fertility rates (ASFRs) of a given period and if they were not subject to mortality^15^. The ASFR represents the number of births per 1000 women for a specific age group in the past year. TFR was counted as children per woman calculated by summing the single-year ASFRs in a given period, and it is considered to be “very low” if it falls below 1.5 and “lowest-low” if below 1.3^16,17^.

#### Estimated TFRs

The estimated TFRs referred to the annual TFRs estimated by using the 2020 NPCC data in this study. The 2020 NPCC provided the number of births to women of different ages and age-specific women populations at national, urban–rural, and regional levels in the year 2020. Therefore, the estimated TFRs overall and at different levels in 2020 could be directly extracted from the 2020 NPCC.

Considering the underreported issues in the official statistic system before 2020, we used scaled data from the 2020 NPCC and some structural data from censuses and sample data between 2000 and 2019 to estimate previous TFRs. To assess national TFRs from 2000 to 2019, we applied the reverse survival method^18^ to the overall population aged 1–20 years old (n = 175,077,370 boys and n = 153,565,998 girls) and the female population aged 16–70 years old (n = 505,951,153) obtained from the 2020 NPCC. The process of applying the reserve survival method to estimate previous TFRs is shown as follows:

(1) Estimate the number of births in each year preceding 2020.

We divided the population aged 1–20 years old in 2020 by using life table survivorship ratios^18^ and cohort survival probabilities^18^ to calculate the annual number of births before 2020. The survivorship ratio was expressed as

1 $${P}_{x,t}=\frac{{L}_{x,t}}{{L}_{x-1,t-1}},$$

and the cohort survival probability was expressed as

2 $${S}_{0,t}=\frac{{L}_{0,t}}{{l}_{0,t}}.$$

In Eqs. (1) and (2), the ${L}_{x,t}$ is the number of person-years of the population aged *x* years in the life table living from *x* years to (*x* + 1) years in year *t*, and the ${l}_{0,t}$ is the initial population in the life table. The parameters ${L}_{x,t}$ and ${l}_{0,t}$ were extracted from the Coale–Demeny West Pattern model life tables with life expectancy at birth. We included the life expectancy at birth in 2000, 2005, 2010, 2015, and 2020 provided by China NBS, and we calculated the life expectancy at birth of intermediate years by using the linear interpolation. The life expectancy at birth by sex for each year from 2000 to 2020 is displayed in Appendix S2.

The populations aged 1–20 by single years of age in 2020 were first divided by survivorship ratios to estimate the population aged 0 from 2000 to 2019, which was algebraically expressed as

3 $${N}_{0,t}=\frac{{N}_{x,2020}}{\left[\left(\frac{{L}_{x,2020}}{{L}_{x-\mathrm{1,2019}}}\right)*\left(\frac{{L}_{x-\mathrm{1,2019}}}{{L}_{x-\mathrm{2,2018}}}\right)*\cdots *\left(\frac{{L}_{1,t+1}}{{L}_{0,t}}\right)\right]}.$$

In Eq. (3), the ${N}_{x,t}$ is the population aged *x* in year *t*, and *x* plus *t* equals 2020.

Then, the populations aged 0 were divided by cohort survival probabilities to calculate the annual number of births in each year preceding 2020, according to the formula:

4 $${B}_{t}=\frac{{N}_{0,t}}{\frac{{L}_{0,t}}{{l}_{0,t}}}.$$

In Eq. (4), the ${B}_{t}$ is the number of births in year *t*.

The estimations of births were implemented separately by sex. Afterwards, we summed the number of births by sex to obtain the total number of births from 2000 to 2019.

(2) Calculate the number of births to women of different ages in each year preceding 2020.

We adopted age-specific fertility distributions^18^ to segment the total number of births into the number of births to women of different ages in each year preceding 2020. The age-specific fertility distributions in 2000, 2010, and 2020 were obtained from the 2000, 2010, and 2020 NPCCs, respectively. As structural data, they were hardly affected by underreporting. The distributions in intercensal years were derived from the estimations by linear interpolations between every two censuses. The age-specific fertility distributions in 2000, 2010, and 2020 are displayed in Appendix S3 and Appendix S4 displays the numbers of births to women of different ages (in thousands) in different years estimated from the data in the 2020 national population census of China.

(3) Calculate the number of women by age in each year preceding 2020.

Women of different ages between 16 and 70 in 2020 were divided by survivorship ratios to determine the populations of women of different ages between 15 and 49 in the years preceding 2020, according to the formula:

5 $${N}_{x,t}=\frac{{N}_{y,2020}}{\left[\left(\frac{{L}_{y,2020}}{{L}_{y-\mathrm{1,2019}}}\right)*\left(\frac{{L}_{y-\mathrm{1,2019}}}{{L}_{y-\mathrm{2,2018}}}\right)*\cdots *\left(\frac{{L}_{x+1,t+1}}{{L}_{x,t}}\right)\right]}.$$

In Eq. (5), the $t-x$ equals $2020-y$.

We then averaged the populations of women between two adjacent years to generate the number of women as denominators of age-specific fertility rates (ASFRs). The numbers of women by age (in thousands) in different years estimated from the data in the 2020 national population census of China are displayed in Appendix S5.

(4) Calculate the ASFRs and total fertility rates (TFRs) preceding 2020.

After obtaining the number of births to women of different ages and the number of women by age in each year, we could generate annual ASFRs by the formula:

6 $${f}_{x}=\frac{{B}_{x}}{{W}_{x}}\times 1000\mathrm{\textperthousand}.$$

In Eq. (6), the ${f}_{x}$ is the fertility rate of women aged x, ${B}_{x}$ is the number of births to women aged x, and ${W}_{x}$ is the number of women aged x.

Then, TFR could be calculated by adding up the ASFRs according to the equation:

7 $$TFR=\sum {f}_{x}.$$

Since the census data including all the residents of the corresponding age and gender in China were used for analyses, no 95% confidence intervals (CIs) were reported.

#### Estimated rural–urban and regional TFRs

According to the NBS, urban areas refer to the areas under the jurisdiction of neighborhood committees (“Juweihui” in Chinese), while rural areas encompass regions governed by village committees (“Cunweihui” in Chinese). To calculate the estimated rural–urban TFRs from 2000 to 2019, we first included the annual urbanization rate, referring to the share of population residing in the urban areas and as a resulting indicator of rural-to-urban migration, of women by age and birth numbers from the NBS of China before 2020. Next, we separated the age-specific numbers of women and their birth numbers from 2000 to 2019 estimated previously by the reverse survival method into rural and urban groups. Then, we transformed annual rural and urban birth numbers into age-specific patterns using rural and urban age-specific fertility distributions, respectively. Finally, we calculated the annual rural and urban ASFRs and TFRs before 2020 based on the above estimations.

We used the Chinese standard geographical classification of seven regions in mainland China: north, northeast, east, central, south, southwest, and northwest. The TFRs by region and regional rural–urban location were calculated by using the same method as above.

#### Reported TFRs

The reported TFRs, which were considered to exist underreporting issues, represented those directly obtained from NPCCs and population sample surveys conducted and published by the NBS of China before 2020.

**Figure 3:**
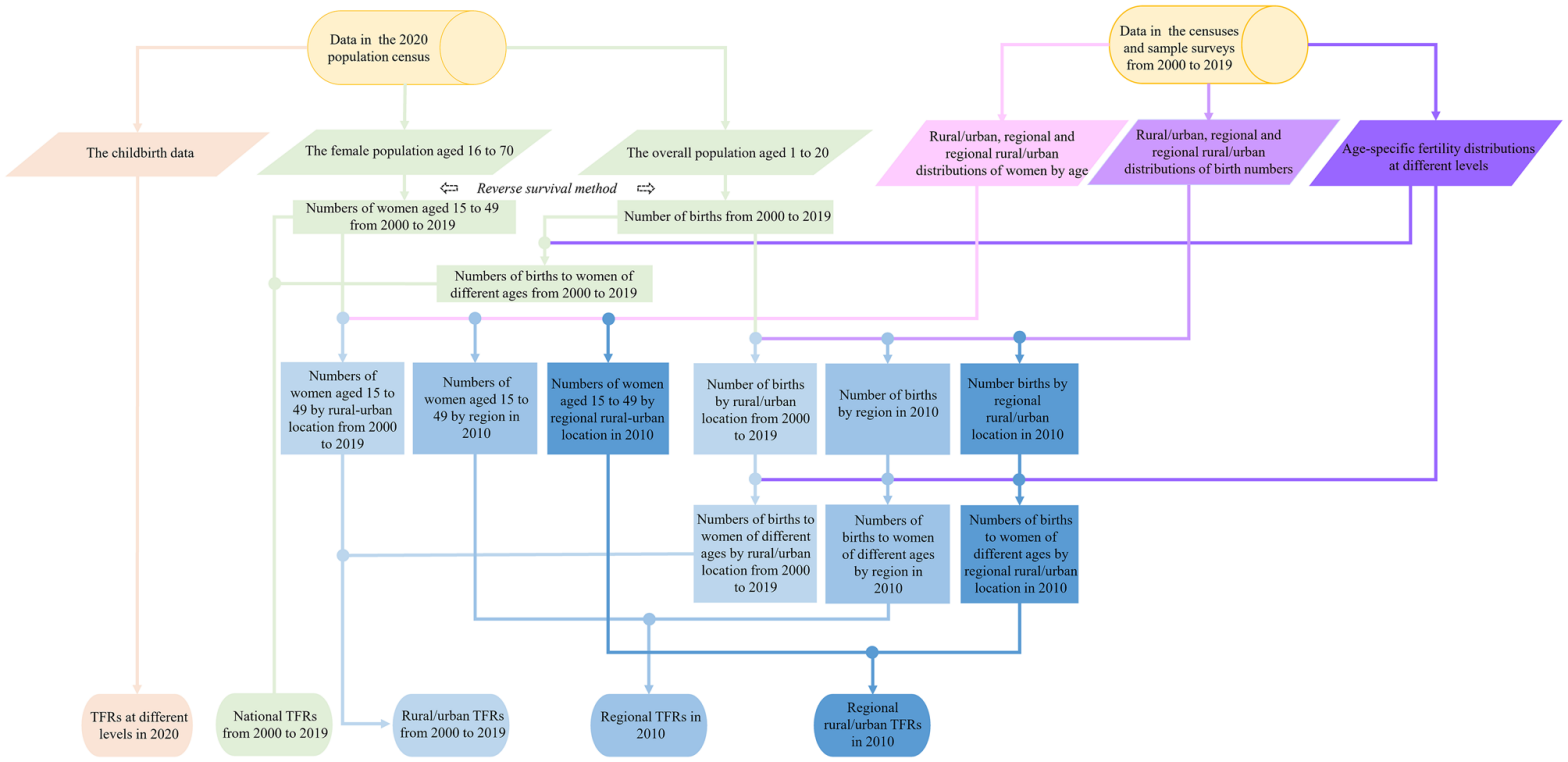
Process of estimations of total fertility rates (TFRs) in different years at different levels. The 2000 and 2010 censuses were used to provide some structural data including age-specific fertility distribution at different levels and rural/urban, regional, and regional rural/urban distributions of women by age and birth numbers to assist in estimating TFRs. The rural/urban distribution is known as the urbanization rate. The 1%/1‰ sample surveys were used to provide structural data on the urbanization rate from 2001 to 2019 (excluding 2010) to assist analyses.

The detailed process to calculate estimated and reported TFRs in different years at different levels is shown in Fig. 3.

#### Data analysis

We first presented and compared the trends in estimated and reported TFRs from 2000 to 2020 at the national level. Next, the trends in the estimated rural and urban TFRs were also examined, and we also calculated the trends in differences between the estimated rural and urban TFRs in each year. Finally, the changes in estimated regional TFRs between 2010 and 2020 were calculated and displayed, stratified by rural–urban location.

### Ethics approval and consent to participate

All methods were performed in accordance with relevant guidelines and regulations. All analyses were conducted based on published aggregated data, and ethical approval or informed consent was not required.

## Results

### Gaps between newly estimated and reported TFRs

**Figure 4:**
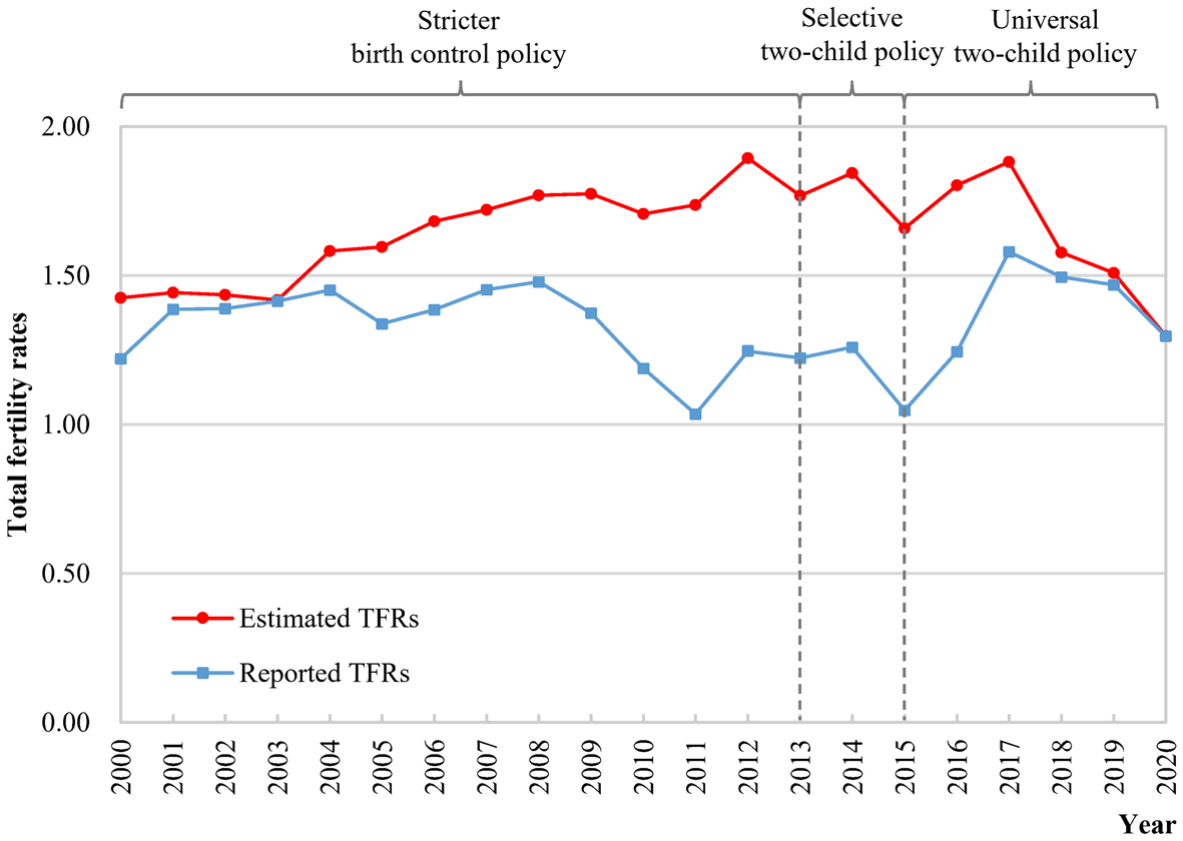
National trends in the estimated and reported TFRs in China, 2000–2020.

Overall, there were significant gaps between the estimated and reported TFRs before 2020, and the estimated TFRs based on the 2020 NPCC remained higher than the reported TFRs from the previous government statistics (Fig. 4 and Appendix S6). The gaps in TFRs reached up to 0.5 from 2010 to 2016 and gradually narrowed after 2017. The estimated TFRs have fluctuated above the very low fertility rate (TFR = 1.5) in the past two decades and have shown a wavelike decline in the past decade. Four years had an apparent increase compared with the last year: 2012 (TFR = 1.89), 2014 (TFR = 1.84), 2016 (TFR = 1.80), and 2017 (TFR = 1.88). The estimated TFRs showed an apparent drop in 2015, 2018, and 2020, with a decrease of more than 10% compared with the last year.

The average of the estimated TFRs from 2000 to 2020 was 1.64, close to the average number of children ever born by some particular cohorts of women who largely completed their fertility histories (Table 2). From 2000 to 2019, the number of annual births estimated from the 2020 NPCC remained above 14 million and even exceeded 18 million in several years, including 2009, 2011 to 2014, and 2016 to 2017 (Appendix S6).

**Table 2:** The average number of children ever born by some particular cohorts of women.

Birth cohorts of women	Age of women in 2020	The average number of children ever born to women
1985	35	1.56
1984	36	1.60
1983	37	1.60
1982	38	1.60
1981	39	1.62
1980	40	1.63
1979	41	1.61
1978	42	1.61
1977	43	1.60
1976	44	1.60
1975	45	1.60
1974	46	1.60
1973	47	1.59
1972	48	1.59
1971	49	1.59

The data were extracted from the 2020 national population censuses of China.

### Trends in differences between estimated rural and urban TFRs

**Figure 5:**
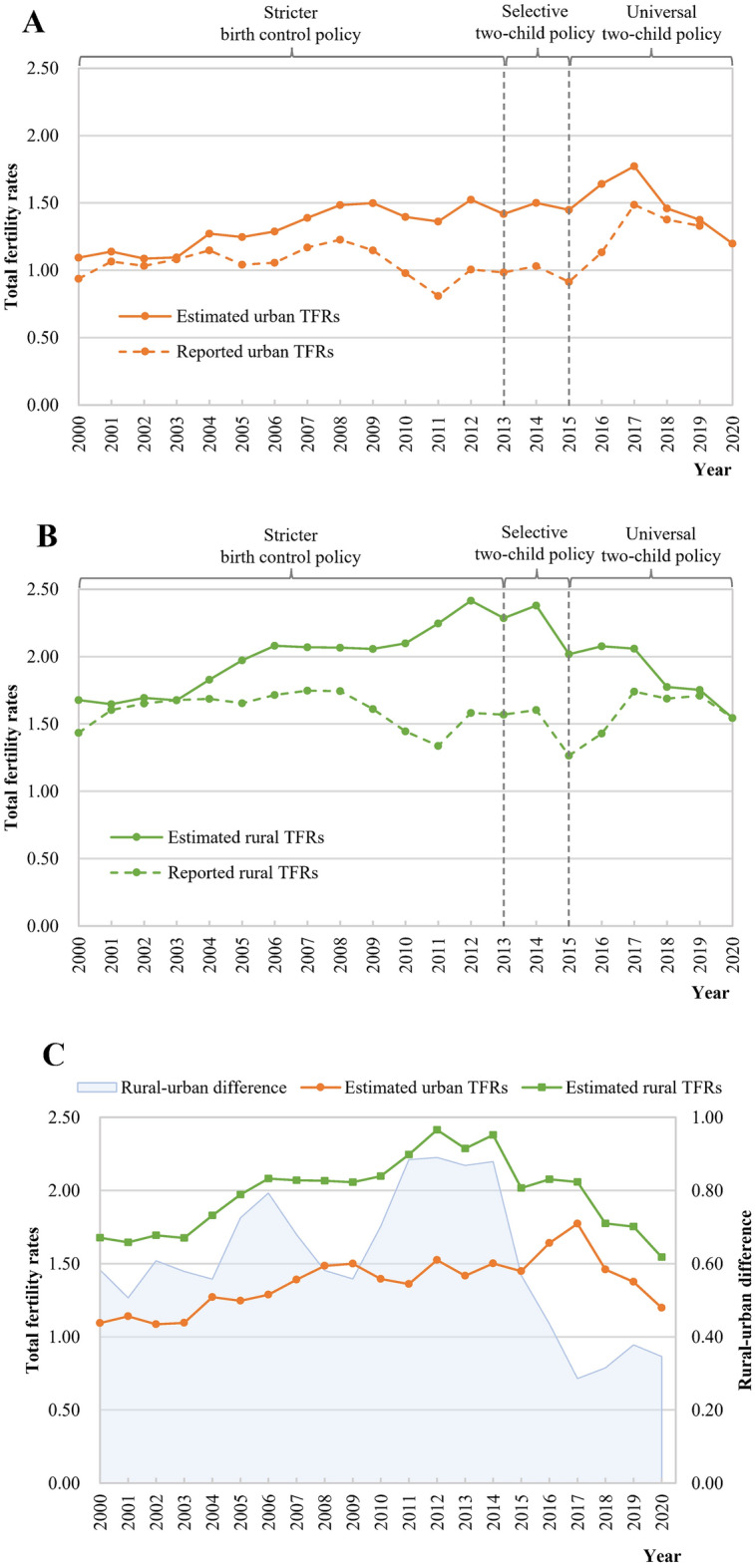
The estimated TFRs by rural–urban location in China, 2000–2020.

In general, the rural–urban differences in the estimated TFRs decreased, especially after 2014. The estimated TFRs fluctuated below 1.5 in urban areas compared to above 1.8 in rural areas (Fig. 5 and Appendix S7). The estimated rural TFRs exceeded the replacement level of approximately 2.1 from 2011 (2.25) to 2014 (2.38). The estimated urban and rural TFRs showed homologous fluctuation modes, especially with rebounds in 2012, 2014, and 2016. However, in contrast to the estimated rural TFRs, the estimated urban TFR showed a larger rebound in 2016 (an increase by 13.30% in urban areas vs an increase by 2.97% in rural areas) and continued to rise in 2017 (an increase by 8.02% in urban areas *vs* a decrease by 0.87% in rural areas). The differences between the estimated rural and urban TFRs demonstrated an overall increasing trend from 0.58 in 2000 to 0.88 in 2014, but they declined afterwards from 0.88 in 2014 to 0.35 in 2020. In both urban and rural areas, the estimated TFRs remained higher than the reported TFRs over the study period, and the gaps between the estimated and reported TFRs were larger in rural areas than in urban areas (Fig. 5 and Appendix S7).

### Differences in regional and regional rural–urban fertility between 2010 and 2020

**Figure 6:**
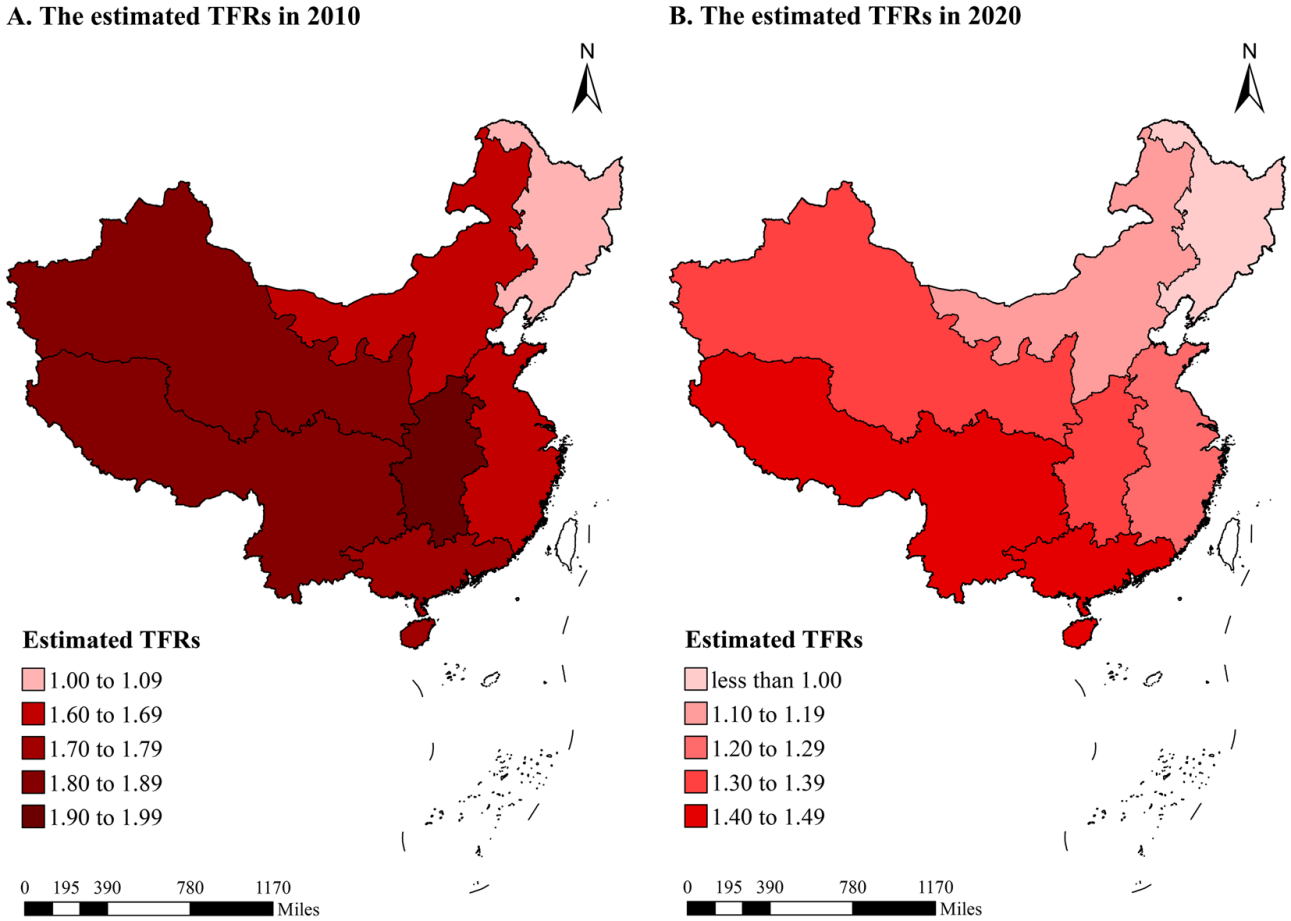
The estimated TFRs by region in China between 2010 and 2020.

The TFRs declined in all regions between 2010 and 2020, while the extents varied across regions (Fig. 6 and Appendix S8). Obvious decreases in TFRs by over 25% occurred in the north, east, central, and northwest regions, and among them, the central region experienced the largest decline by 30.57%. The number of regions below the threshold of the lowest-low fertility (TFR = 1.3) increased from only one (northeast) in 2010 to three (northeast, north, and east) in 2020. The northeast region continued to have the lowest fertility level in both 2010 (TFR = 1.07) and 2020 (TFR = 0.86). For regions with the highest fertility level, we found a transition from the central region in 2010 (TFR = 1.91) to the southwest and south regions in 2020 (TFRs = 1.40 and 1.49).

**Figure 7:**
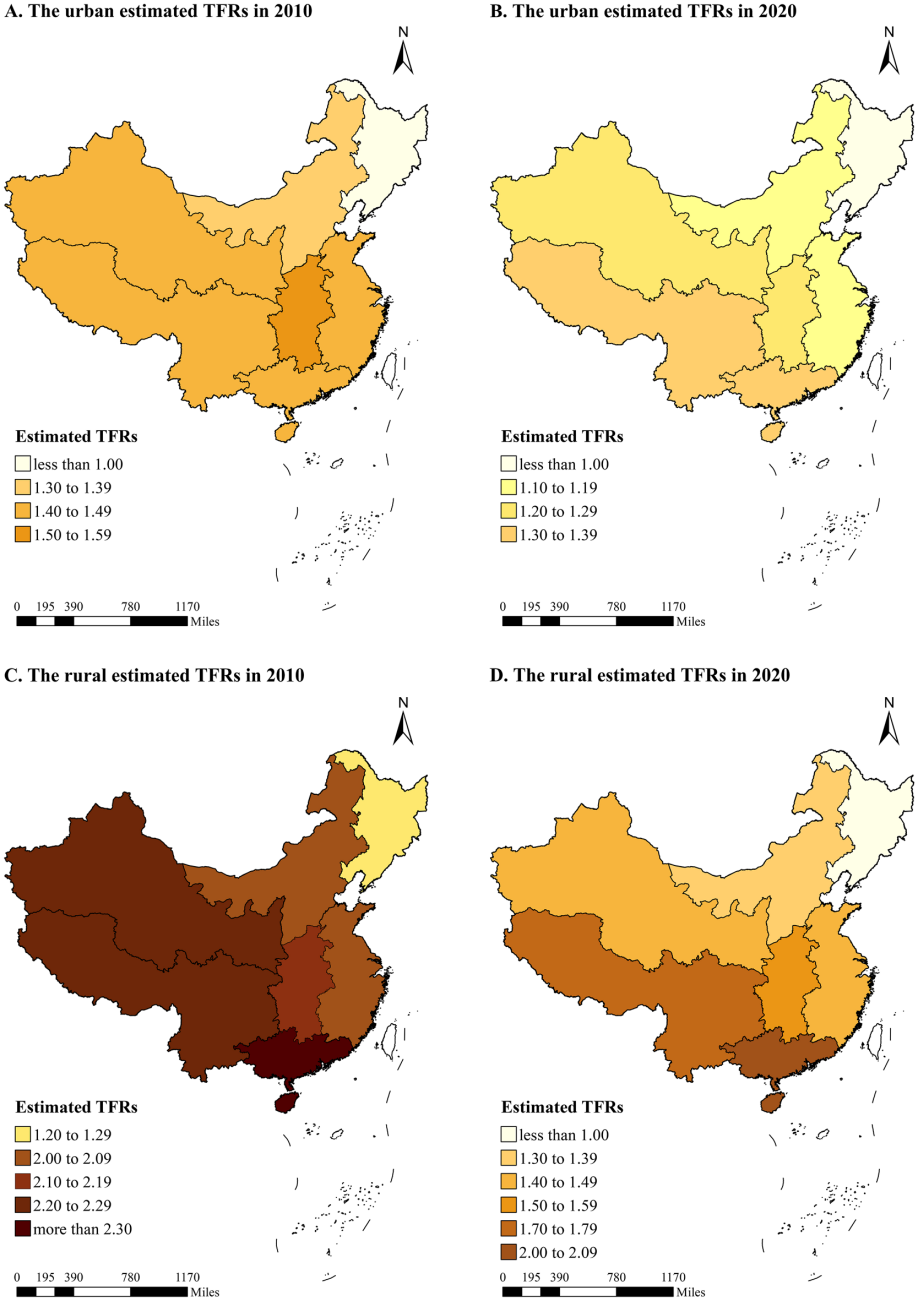
The estimated TFRs by regional rural–urban location in China between 2010 and 2020.

The estimated regional rural–urban TFRs in 2010 and 2020 were mapped in Fig. 7 and exhibited in Appendix S8. Women living in rural areas had a higher average number of births than their urban counterparts in all regions in both census years, except for the northeast region in 2020. The rural–urban differences in fertility levels declined between 2010 and 2020 in all regions, with a decrease of more than 70% in the northeast, northwest, and north regions. A decrease in estimated TFRs was also observed between 2010 and 2020 in all regional urban and rural areas, with rural areas experiencing larger decreases in all regions (Appendix S8).

### Robustness checks

We further changed the combination of age-specific fertility distributions to conduct robustness checks. We adopted age-specific fertility distributions in 2000, 2005, 2010, 2015, and 2020, where data in 2005 and 2015 were respectively extracted from the 1% sample survey in 2005 and 2015, and the rest were obtained from censuses. The fertility distributions of other years were calculated by linear interpolation of the fertility distributions in these 5 years. The TFRs from robustness checks are very close to the previously estimated TFRs (Appendix S9).

## Discussion

The fertility levels of China have been controversial in the recent two decades, and the 2020 NPCC offers an opportunity to assess TFRs to provide more accurate estimations based on the best data ever. To our knowledge, this study was the first to use the 2020 NPCC data to estimate TFRs and examine rural–urban and regional differences in TFRs from 2000 to 2020 in China. Our results indicated that the reported TFRs in China were highly likely to be underreported, and there were significant gaps between newly estimated TFRs and reported TFRs over time. The estimated TFRs remained above 1.5 children per woman during 2004–2019 and rebounded in the years after the birth control policy changes. Additionally, rural areas had higher estimated TFRs than urban areas, but the rural–urban differences in the estimated TFRs started to narrow quickly after 2014. Regarding geographic regional differences, in 2020, the southwest and south regions had the highest estimated TFRs (1.40–1.49), followed by the northwest (1.30) and central regions (1.39), and the northeast region had the lowest estimated TFR (less than 1.00). The estimated TFRs in all regions displayed a decline between 2010 and 2020, especially in rural areas, and the estimated TFRs in the rural north, east, central, and northwest areas even experienced a cliff-like decline. These findings have implications for dynamic changes in health and population policies.

In China, major sources of fertility data included NPCCs, intercensal sample surveys (including 1% sample surveys and 1‰ sample surveys), household registration systems, family planning statistics, and national retrospective fertility surveys^10^. These data sources were collected through different channels to serve different purposes, but all of them faced the challenges of underreporting issues, especially for infants who were born near the data collection period^10^. The 2020 NPCC adopted data innovations beyond the traditional collection methods to reduce underreporting and improve accuracy^14^. In particular, the 2020 NPCC used the ID number to verify and compare information across databases, which effectively reduced re-reporting and omission in the context of massive population migration.^14^ The quality of the data made us believe that our estimations were more accurate and were closer to the real TFRs in China, and the underreporting data contributed to the relatively low reported TFRs.

**Figure 8:**
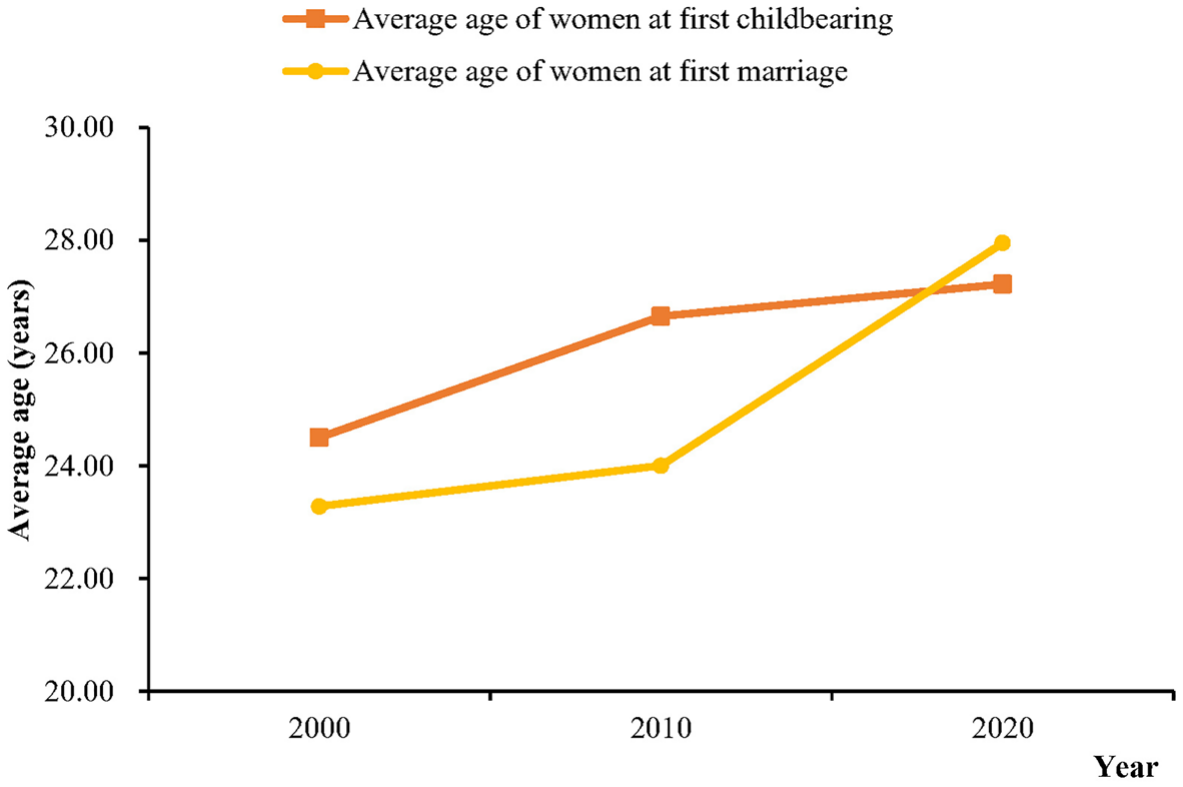
The average age of women at first marriage and first childbearing in 2000, 2010, and 2020. The data were extracted from the 2000, 2010, and 2020 national population censuses of China.

This study indicated that the official statistics might have previously underestimated and underreported TFRs in China. Our estimations were in line with existing studies with conclusions of higher births^19^. Despite TFRs above 1.5 found in this study from 2000 to 2020, China still has a great risk of a declining and very low fertility level. One study stated that if China insisted on the universal two-child policy, the overall TFR would reach 1.81 in 2030^20^. However, the trend in China’s TFRs was not that optimistic based on our estimates, which has gradually declined since 2018. The first possible explanation for decreasing TFRs is the significant delays in marriage and childbirth (Fig. 8). Since the late 1990s, the expansion of higher education has brought about a substantial increase in women’s education level^21^. Similar to Europe, delayed childbearing partially resulted from the wider accessibility of tertiary education and the rising age of graduation in China^22^. Second, China is experiencing a transition in fertility preferences, and couples are choosing to remain childless or to have fewer children^10,23^. With the rapid development of social security, the traditional Chinese concept of “more children bring more blessings” has drifted away. In addition, young adults face the structural cost of living issues, including high costs of time, childrearing, and education^24^. Also, we cannot ignore the impact of COVID-19 on the 2020 fertility rates in China, and a steep drop in the fertility rate was detected after the COVID-19 pandemic in some high-income countries^25^.

We also observed that rural–urban differences in the estimated TFRs have shrunk in recent years. The birth control policy consists of a set of regulations, including the spacing of children (in cases in which second children are permitted)^26^. The one-child policy was strictly enforced for urban residents. In rural areas, it soon became clear that a one-child rule was unenforceable because of the importance of labor capital in the family-based agrarian economy, dependence on children in old age, and the tradition of son preference^27^. In addition, entering the twenty-first century, China's rapid economic development has led to significant improvements in rural areas, and the process of economic growth generates sufficient resources so that households can rear more children while still providing the desirable amount of education investment per child^28^. Therefore, the increase in TFRs in rural areas from the beginning of the twenty-first century until the implementation of the two-child policy has been significant. The implementation of the universal two-child policy had short- and long-term effects on China’s TFRs, especially for urban areas. Some studies indicated that in addition to the prevalence of having additional children, the TFR of second children in rural areas was far higher than the TFR of second children in urban areas before 2016^29^. The universal two-child policy did not have an impact on the TFR of the third and more child of the urban census registered population, because it was still illegal for the urban census registered population to have a third child^29^. In addition, the relatively strict implementation of the one-child policy before the policy adjustment resulted in the accumulation of a large target group for the two-child policy in urban areas, which drove up the TFRs significantly; in rural areas, where the "one-and-a-half-child" policy was implemented, the target group for the two-child policy was basically confined to women of childbearing age who gave birth to boys, and the size of the target group was relatively smaller. Therefore, the implementation of the universal two-child policy mainly affects the TFR of second children in urban areas, but it was not a sufficient engine for long-term fertility growth. However, there was a trend in that convergence of rural TFR toward the urban level in China, which was consistent with other developing countries in the world^30^. Two reasons may contribute to the decreasing differences in rural–urban TFRs. The first is the convergence of childbearing willingness between rural and urban areas. Rural and urban residents in the eastern coastal areas had a similar preference for the number of children^31^, and even in less-developed western areas, only a small proportion of rural dwellers preferred three or more children in China^20^. Given the increased rearing costs and greater importance attached to childhood education, both urban and rural parents face similar barriers to childrearing^24^. The second one is the convergence of childbearing time between rural and urban areas. The migration of rural populations leads to their childbearing time being delayed. Moreover, the improvement in educational attainment and reduction in the rural–urban education gap also displayed childbearing time delays in rural areas^32^. In addition, some studies have reported that the earlier and more sustained decline in urban fertility in developing countries is accompanied by a more pronounced decline in rural fertility as society develops, and the decline of fertility is more moderate in the later stages of social development  and transition in urban areas^30^. Taken together, the fertility patterns in rural areas have moved closer to those in urban areas, and relying solely on rural fertility levels was not expected to significantly increase the TFR in China.

The decreased estimated TFRs in all seven regions of China between 2010 and 2020 presented a huge challenge. It was suggested that regional variations in fertility levels mainly resulted from differences in economic structure, cultural tradition, and policy implementation^33,34^. Our study indicated that the estimated TFRs in the southwest and south regions were the highest in 2020, and overtook the central regions. First, the southwest and south regions possess a high proportion of ethnic minority residents. The Chinese government allowed minority autonomous regions to formulate or modify their marriage laws and family planning policies in the context of local customs. For instance, a third child or getting married two years earlier is allowed for some ethnic minorities^26^. Second, the socioeconomic, demographic, and cultural factors all play important roles in determining the TFRs. Southern China has a preference for multiple children or boys compared to other regions^35^, so with the gradual relaxation of the birth control policy, the TFRs in the southwestern and southern regions have gradually overtaken that of the central region compared to 2010. In addition, drawing on past pandemics, scholars have suggested that the COVID-19 pandemic will bring about fertility decline^25^. China's COVID-19 started in Hubei, an important province in the center of the country, and the closer to the epicenter of the outbreak, the more severe the epidemic^36^. As a result, the TFRs in central China will decline in 2020. The estimated TFR in the northeast region was the lowest, which was consistent with previous studies^37^. This may be attributed to the earliest regions to industrialize, population migration, and relatively poor economic development levels in recent years^38^. It was also noticed that compared to 2010, the variations in the estimated TFR in 2020 decreased in all regions, further indicating the risk of low fertility levels all over China.

The fluctuations in estimated TFRs during 2000–2020 clearly demonstrated the impacts of fertility policies. Starting in the 1970s, the birth control policy was designed to develop the economy and improve living standards. Subsequently, in 2013 and 2015, the selective two-child and universal two-child policies were implemented, respectively. Our findings of the relatively higher TFRs after 2014 were in line with other studies of the two-child policy’s effects in China^19,39^. There was a trough in 2015 and 2018, in which the demands were largely released in 2014 and 2016–2017, respectively, immediately after the enactment of these policies. Some studies indicated that most of women of child-bearing age in the policy adjustment year, who were restricted from having a second child by China's previous stricter birth control policy but wanted to have a second child, would give birth to a second child in two years after implementation of the new fertility policy^40^. Recent data on births indicated a further decline^42^, and in 2021, a three-child policy was implemented quickly to cope with the risk of decreasing fertility levels, and further studies will be needed to examine its impact on TFRs.

Our studies showed that the two-child policy was related to a moderate and temporary increase in TFRs, but its impact has been extremely short-lived and has not been effective in curbing the overall trend of declining fertility levels. Therefore, in addition to the relaxation of the birth control policy, our results suggest that it is important to take comprehensive measures to stop the increasing risk of low fertility levels and help young people reduce childrearing barriers. ﻿Empirical evidence from low-fertility countries indicates that the implementation of fertility support policies, encompassing economic support policies (e.g. family allowances, maternity- and paternity-leave benefits, and tax breaks), service support policies (e.g. childcare services, maternal health care services, and educational input), and time support policies (e.g. maternity leave, paternity leave, and parental leave), has proven in boosting TFRs to some extent^43–45^. According to surveys conducted by the National Health Commission, the average number of children planned by Chinese families in 2021 was 1.64^46^. This data suggests a notable potential for a substantial increase in China's fertility rate if an effective fertility support system is established. First, the government should tackle measures that decrease the direct and opportunity costs of childbearing, such as providing sufficient parental leave, expanding available and qualified childcare facilities, regulating the price of childcare products and services, providing adequate career opportunities for rural residents to reduce economic and childrearing costs, and encouraging the development of early education institutions. Second, Chinese society from all regions and rural–urban perspectives should transmit the fertility thinking and make the family-planning policy more inclusive, such as making cities more family-friendly, improving work-life balance for females, helping them juggle work and childcare, and providing subsidies, tax relief and rewards for families with two or more children.

This is the first study to apply the reverse survival method to the 2020 NPCC data to assess trends in TFRs and its rural–urban and regional differences in China. The method has been well established to produce estimates that are consistent and hardly affected by erroneous assumptions^47^. In addition, compared to other major sources of the fertility data including NPCCs, intercensal sample surveys, household registration systems, family planning statistics, and national retrospective fertility surveys^10^, all of which faced the challenges of underreporting issues, the 2020 NPCC adopted data innovations beyond the traditional collection methods to reduce underreporting and improve the accuracy^14^. In particular, the 2020 NPCC used the ID number to verify and compare information across databases, which effectively reduced re-reporting and omission in the context of massive population migration^14^. The quality of the data made us believe that our estimations were more accurate and were closer to the real TFRs in China, and the underreporting data contributed to the relatively low reported TFRs. The findings from the reassessment of TFRs provided new evidence on the accurate fertility levels in the past two decades in China, and it helped better understand different roles of the family-planning program and the socioeconomic transformation in driving the fertility and population changes. Most importantly, they could help better project the fertility and population size in future, which has the great significance to establish fertility- and health-related policies in China and has inspiration for other low- and middle-income countries to improve family-planning programs.

There are several limitations to note. First, we interpolated to estimate the fertility schedules for individual years owing to unavailable age-specific fertility distributions in each year. Although many researchers believed that the effect remained limited, this limitation can affect the estimates of TFR^8^. Second, due to the data were lacking on the census life tables calculated by the NBS of China, we used the model life tables as the standard to estimate the survivorship for children and women. As only a small minority of children and women died during the 20 years, the impact of errors in the estimates of survival probabilities would be little.

## Conclusions

This study provides more reliable TFR estimates and their rural–urban and regional differences from 2000 to 2020 by using the newly released high-quality 2020 NPCC data in China. Our findings indicated that although China’s TFRs were not as low as those previously reported by the official statistics in the past 20 years, China still has a greater risk of a very low fertility level in all national, regional, and rural–urban levels. Therefore, in addition to relaxing birth restrictions and policies, it is more important to adopt comprehensive strategies to improve social supportive policies and services, including reducing the costs of marriage, childbearing, and child education, as well as fostering work-family balance, to increase fertility levels.

### Supplementary Information

Supplementary Information.

## Data Availability

All data in this study can be made available upon request to the corresponding authors by email: rzjing@ruc.edu.cn or he.zhu@pku.edu.cn.
