# Supplementary material for: A reassessment of trends and rural–urban/regional differences in the total fertility rate in China, 2000–2020: analyses of the 2020 national census data
Source: Sci Rep. 2024 Apr 13;14:8601. doi: 10.1038/s41598-024-59177-2 (PMC11016060; doi:10.1038/s41598-024-59177-2)
Supplement: Supplementary file 1 — Supplementary Information. [file 41598_2024_59177_MOESM1_ESM.docx]

**Supplementary appendix**

Content

[***Appendix 1:*  Information technology applications to improve the data quality in the 2020 national population census of China** 2](#_Toc158072772)

[***Appendix 2:* Life expectancy at birth by sex for each year from 2000 to 2020** 3](#_Toc158072773)

[***Appendix 3:* Age-specific fertility distributions in 2000, 2010, and 2020** 4](#_Toc158072774)

[***Appendix 4:* Numbers of births to women of different ages (in thousands) in different years estimated from the data in the 2020 national population census of China** 5](#_Toc158072775)

[***Appendix 5:*** 7](#_Toc158072776)

[**Numbers of women by age (in thousands) in different years estimated from the data in the 2020 national population census of China** 7](#_Toc158072777)

[***Appendix 6:* Trends in the estimated and reported total fertility rates (TFRs) and the estimated number of annual births from 2000 to 2020 in China** 9](#_Toc158072778)

[***Appendix 7:* Trends and differences in the reported and estimated total fertility rates (TFRs) between rural and urban areas from 2000 to 2020 in China** 10](#_Toc158072779)

[***Appendix 8:* Comparison of estimated total fertility rates (TFRs) between 2000 and 2020, stratified by region and urban-rural areas** 11](#_Toc158072780)

***Appendix 1:*  Information technology applications to improve the data quality in the 2020 national population census of China**

Compared with previous censuses, information technologies were fully adopted in the 2020 national population census of China to innovate the data collection methods and improve the data quality. *First*, enumerators used electronic equipment to collect information, and the data could be timely verified by 800 logical review rules set in the digital program to directly transmit to the national unified cloud platform set by statistical bureaus. Therefore, the new collection can effectively avoid possible misstatement, loss, alteration, and other problems in the process of data recording, transmission, and summary. *Second*, an online registration channel was introduced to respondents, and they were enabled to fill out and submit the questionnaire themselves by scanning the QR code, which reduced the workload and enhanced the cooperation of the public. *Third*, online centralized management was carried out to supervise the work of 7 million census enumerators in real-time. The locations, sounds, registration time, and registration contents of enumerators were supervised during the investigation, which can help distinguish abnormal behaviors of enumerators and thus improve the quality of data collection. *Fourth*, big data were used to help distinguish the residence of respondents. For instance, big data from electricity meters were used to judge whether people living in a house, and big data from mobile phones were used to ascertain the usual residence of migrants.

***Appendix 2:*** **Life expectancy at birth by sex for each year from 2000 to 2020**

| **Year** | **Life expectancy at birth for a man** | **Life expectancy at birth for a woman** |
| --- | --- | --- |
| 2000 | 69.63 | 73.33 |
| 2001 | 69.87 | 73.71 |
| 2002 | 70.11 | 74.10 |
| 2003 | 70.35 | 74.48 |
| 2004 | 70.59 | 74.87 |
| 2005 | 70.83 | 75.25 |
| 2006 | 71.14 | 75.67 |
| 2007 | 71.45 | 76.10 |
| 2008 | 71.76 | 76.52 |
| 2009 | 72.07 | 76.95 |
| 2010 | 72.38 | 77.37 |
| 2011 | 72.63 | 77.78 |
| 2012 | 72.88 | 78.19 |
| 2013 | 73.14 | 78.61 |
| 2014 | 73.39 | 79.02 |
| 2015 | 73.64 | 79.43 |
| 2016 | 73.99 | 79.72 |
| 2017 | 74.33 | 80.01 |
| 2018 | 74.68 | 80.30 |
| 2019 | 75.02 | 80.59 |
| 2020 | 75.37 | 80.88 |

***Appendix 3:* Age-specific fertility distributions in 2000, 2010, and 2020**

| **Age in years** | **Year 2000 (%)** | **Year 2010 (%)** | **Year 2020 (%)** |
| --- | --- | --- | --- |
| 15 | 0.01 | 0.01 | 0.05 |
| 16 | 0.05 | 0.06 | 0.13 |
| 17 | 0.21 | 0.25 | 0.27 |
| 18 | 0.54 | 0.66 | 0.49 |
| 19 | 1.44 | 1.34 | 0.89 |
| 20 | 3.82 | 3.58 | 1.52 |
| 21 | 6.82 | 5.61 | 2.28 |
| 22 | 8.57 | 6.92 | 3.08 |
| 23 | 10.24 | 8.70 | 4.09 |
| 24 | 11.32 | 8.24 | 5.18 |
| 25 | 10.65 | 7.07 | 5.89 |
| 26 | 9.30 | 6.84 | 6.75 |
| 27 | 7.74 | 6.48 | 7.60 |
| 28 | 6.44 | 6.98 | 7.85 |
| 29 | 5.50 | 5.43 | 8.54 |
| 30 | 4.57 | 4.48 | 8.60 |
| 31 | 3.75 | 4.08 | 7.36 |
| 32 | 2.72 | 3.53 | 6.51 |
| 33 | 1.71 | 2.72 | 5.32 |
| 34 | 1.30 | 2.61 | 3.99 |
| 35 | 0.93 | 2.28 | 3.10 |
| 36 | 0.69 | 2.08 | 2.50 |
| 37 | 0.50 | 1.78 | 2.08 |
| 38 | 0.28 | 1.51 | 1.72 |
| 39 | 0.17 | 1.23 | 1.08 |
| 40 | 0.13 | 1.12 | 0.85 |
| 41 | 0.10 | 0.79 | 0.60 |
| 42 | 0.10 | 0.76 | 0.41 |
| 43 | 0.08 | 0.52 | 0.30 |
| 44 | 0.07 | 0.49 | 0.22 |
| 45 | 0.06 | 0.46 | 0.17 |
| 46 | 0.05 | 0.43 | 0.15 |
| 47 | 0.05 | 0.46 | 0.14 |
| 48 | 0.04 | 0.35 | 0.14 |
| 49 | 0.03 | 0.19 | 0.14 |

***Appendix 4:* Numbers of births to women of different ages (in thousands) in different years estimated from the data in the 2020 national population census of China**

| Age of women | Year | | | | | | | | | | |
| --- | --- | --- | --- | --- | --- | --- | --- | --- | --- | --- | --- |
|  | 2000 | 2001 | 2002 | 2003 | 2004 | 2005 | 2006 | 2007 | 2008 | 2009 | 2010 |
| 15 | 1.8 | 1.7 | 1.6 | 1.5 | 1.6 | 1.5 | 1.5 | 1.5 | 1.5 | 1.4 | 1.3 |
| 16 | 8.0 | 8.0 | 7.9 | 7.9 | 8.9 | 9.1 | 9.7 | 10.2 | 10.8 | 11.1 | 11.1 |
| 17 | 32.3 | 32.2 | 31.9 | 31.6 | 35.4 | 36.1 | 38.6 | 40.3 | 42.4 | 43.8 | 43.4 |
| 18 | 81.0 | 81.6 | 81.3 | 81.1 | 91.6 | 94.0 | 101.1 | 106.1 | 112.5 | 116.7 | 116.4 |
| 19 | 216.5 | 211.7 | 205.1 | 198.8 | 218.4 | 218.1 | 228.4 | 233.4 | 240.9 | 243.5 | 236.7 |
| 20 | 575.6 | 563.3 | 546.2 | 529.8 | 582.6 | 582.2 | 610.0 | 623.9 | 644.4 | 651.9 | 634.1 |
| 21 | 1027.9 | 994.3 | 952.7 | 912.9 | 991.5 | 978.1 | 1011.4 | 1020.7 | 1039.7 | 1036.9 | 994.1 |
| 22 | 1292.1 | 1247.9 | 1193.6 | 1141.9 | 1237.9 | 1218.9 | 1257.9 | 1266.9 | 1287.7 | 1281.4 | 1225.6 |
| 23 | 1543.8 | 1497.5 | 1438.7 | 1382.7 | 1506.2 | 1490.4 | 1546.2 | 1565.5 | 1600.1 | 1601.6 | 1541.2 |
| 24 | 1706.1 | 1634.5 | 1550.2 | 1469.8 | 1578.6 | 1539.1 | 1572.0 | 1565.9 | 1573.3 | 1546.6 | 1460.1 |
| 25 | 1605.4 | 1527.8 | 1438.7 | 1353.8 | 1442.3 | 1394.0 | 1410.6 | 1391.1 | 1382.5 | 1343.1 | 1251.9 |
| 26 | 1402.2 | 1344.3 | 1275.9 | 1210.7 | 1301.3 | 1269.9 | 1298.2 | 1294.5 | 1301.9 | 1281.2 | 1211.1 |
| 27 | 1167.3 | 1130.7 | 1084.8 | 1041.1 | 1132.4 | 1118.8 | 1158.8 | 1171.3 | 1195.1 | 1194.1 | 1146.9 |
| 28 | 970.9 | 964.1 | 948.4 | 933.4 | 1041.4 | 1055.7 | 1122.1 | 1164.4 | 1220.0 | 1252.1 | 1235.7 |
| 29 | 829.4 | 815.7 | 794.9 | 775.0 | 856.5 | 860.2 | 906.0 | 931.5 | 967.1 | 983.6 | 962.0 |
| 30 | 688.5 | 676.7 | 658.9 | 641.9 | 709.0 | 711.6 | 748.9 | 769.4 | 798.3 | 811.3 | 792.9 |
| 31 | 565.8 | 562.0 | 553.1 | 544.5 | 607.8 | 616.3 | 655.4 | 680.3 | 713.1 | 732.1 | 722.7 |
| 32 | 409.8 | 415.5 | 417.1 | 418.6 | 475.9 | 491.3 | 531.5 | 561.0 | 597.5 | 623.1 | 624.5 |
| 33 | 257.0 | 268.2 | 276.4 | 284.2 | 330.4 | 348.2 | 384.1 | 412.8 | 447.2 | 473.8 | 482.0 |
| 34 | 195.4 | 211.9 | 225.8 | 238.9 | 285.0 | 307.2 | 345.8 | 378.4 | 416.7 | 448.1 | 462.1 |
| 35 | 140.6 | 158.4 | 174.1 | 188.9 | 230.2 | 252.6 | 288.7 | 320.2 | 356.8 | 387.7 | 403.5 |
| 36 | 104.3 | 123.3 | 140.3 | 156.5 | 194.9 | 217.7 | 252.6 | 283.6 | 319.4 | 350.3 | 367.6 |
| 37 | 75.4 | 93.2 | 109.4 | 124.9 | 158.2 | 179.1 | 210.1 | 238.1 | 270.2 | 298.3 | 314.7 |
| 38 | 41.7 | 59.4 | 75.8 | 91.4 | 120.5 | 140.6 | 168.7 | 194.8 | 224.4 | 250.8 | 267.4 |
| 39 | 25.7 | 41.0 | 55.4 | 69.0 | 92.9 | 110.1 | 133.7 | 155.7 | 180.7 | 203.1 | 217.7 |
| 40 | 20.2 | 34.5 | 47.9 | 60.7 | 82.5 | 98.5 | 120.2 | 140.6 | 163.6 | 184.4 | 198.1 |
| 41 | 15.3 | 25.2 | 34.5 | 43.4 | 58.7 | 69.8 | 85.0 | 99.2 | 115.3 | 129.8 | 139.3 |
| 42 | 15.1 | 24.6 | 33.5 | 42.0 | 56.7 | 67.3 | 81.9 | 95.5 | 110.9 | 124.8 | 133.8 |
| 43 | 12.6 | 18.8 | 24.7 | 30.2 | 40.2 | 47.3 | 57.1 | 66.2 | 76.5 | 85.8 | 91.7 |
| 44 | 10.1 | 16.2 | 21.9 | 27.3 | 36.8 | 43.6 | 53.0 | 61.7 | 71.6 | 80.5 | 86.3 |
| 45 | 9.2 | 14.9 | 20.3 | 25.4 | 34.3 | 40.7 | 49.5 | 57.7 | 67.1 | 75.5 | 80.9 |
| 46 | 8.1 | 13.5 | 18.6 | 23.5 | 31.8 | 37.9 | 46.2 | 54.0 | 62.8 | 70.7 | 75.9 |
| 47 | 6.9 | 13.0 | 18.8 | 24.2 | 33.4 | 40.1 | 49.3 | 57.9 | 67.6 | 76.4 | 82.2 |
| 48 | 5.9 | 10.4 | 14.7 | 18.7 | 25.6 | 30.7 | 37.5 | 44.0 | 51.3 | 57.9 | 62.2 |
| 49 | 4.7 | 7.0 | 9.2 | 11.2 | 15.0 | 17.6 | 21.2 | 24.6 | 28.5 | 31.9 | 34.1 |
| Age of women | Year | | | | | | | | | | |
|  | 2011 | 2012 | 2013 | 2014 | 2015 | 2016 | 2017 | 2018 | 2019 | 2020 |  |
| 15 | 2.1 | 3.0 | 3.5 | 4.4 | 4.6 | 5.6 | 6.5 | 6.0 | 6.3 | 5.7 |  |
| 16 | 12.5 | 14.9 | 15.0 | 16.7 | 16.0 | 18.4 | 20.2 | 17.7 | 17.6 | 15.5 |  |
| 17 | 44.8 | 49.1 | 45.9 | 47.8 | 42.9 | 46.5 | 48.4 | 40.4 | 38.3 | 32.2 |  |
| 18 | 116.2 | 123.1 | 111.1 | 111.7 | 96.5 | 100.9 | 101.0 | 81.1 | 73.9 | 59.6 |  |
| 19 | 234.4 | 246.0 | 220.0 | 219.1 | 187.4 | 193.6 | 191.6 | 151.8 | 136.4 | 108.4 |  |
| 20 | 612.2 | 624.8 | 541.6 | 521.1 | 428.7 | 424.1 | 399.2 | 298.7 | 251.1 | 184.4 |  |
| 21 | 957.8 | 975.3 | 843.4 | 809.0 | 663.3 | 653.5 | 612.2 | 455.4 | 380.1 | 276.5 |  |
| 22 | 1185.9 | 1213.2 | 1054.8 | 1018.0 | 840.6 | 835.3 | 790.4 | 595.1 | 504.3 | 373.9 |  |
| 23 | 1495.0 | 1533.9 | 1337.8 | 1295.9 | 1074.6 | 1073.0 | 1021.2 | 774.2 | 661.5 | 495.6 |  |
| 24 | 1440.0 | 1504.7 | 1339.1 | 1326.6 | 1128.0 | 1158.5 | 1138.3 | 895.2 | 798.0 | 628.1 |  |
| 25 | 1261.0 | 1347.3 | 1227.7 | 1247.1 | 1089.1 | 1150.7 | 1165.6 | 947.0 | 874.1 | 714.6 |  |
| 26 | 1238.9 | 1344.8 | 1245.3 | 1285.8 | 1141.7 | 1227.1 | 1264.7 | 1045.8 | 983.1 | 818.6 |  |
| 27 | 1195.3 | 1321.4 | 1245.9 | 1309.5 | 1183.2 | 1293.8 | 1356.3 | 1140.5 | 1089.8 | 922.4 |  |
| 28 | 1281.6 | 1410.2 | 1323.6 | 1385.0 | 1246.2 | 1356.9 | 1416.7 | 1186.6 | 1129.6 | 952.5 |  |
| 29 | 1041.9 | 1193.8 | 1163.8 | 1262.2 | 1174.6 | 1320.4 | 1420.8 | 1224.6 | 1197.9 | 1036.6 |  |
| 30 | 886.9 | 1045.2 | 1044.4 | 1157.7 | 1098.6 | 1256.8 | 1373.9 | 1201.3 | 1190.6 | 1042.7 |  |
| 31 | 799.8 | 934.1 | 926.1 | 1019.6 | 961.7 | 1094.3 | 1190.7 | 1036.7 | 1023.5 | 893.2 |  |
| 32 | 693.7 | 812.8 | 808.1 | 891.9 | 843.1 | 961.3 | 1047.7 | 913.7 | 903.3 | 789.3 |  |
| 33 | 540.9 | 639.2 | 640.2 | 711.0 | 675.9 | 774.4 | 847.7 | 742.1 | 736.3 | 645.5 |  |
| 34 | 498.4 | 568.9 | 552.8 | 597.7 | 554.6 | 621.9 | 667.6 | 574.1 | 560.5 | 484.1 |  |
| 35 | 428.3 | 481.8 | 461.8 | 493.1 | 452.4 | 501.8 | 533.3 | 454.4 | 439.7 | 376.6 |  |
| 36 | 384.2 | 425.9 | 402.7 | 424.3 | 384.4 | 421.4 | 442.8 | 373.2 | 357.4 | 303.1 |  |
| 37 | 327.9 | 362.4 | 341.6 | 359.0 | 324.3 | 354.5 | 371.6 | 312.4 | 298.5 | 252.6 |  |
| 38 | 277.8 | 306.2 | 287.8 | 301.6 | 271.8 | 296.3 | 309.8 | 259.8 | 247.7 | 209.1 |  |
| 39 | 220.2 | 236.4 | 216.5 | 221.0 | 194.0 | 206.0 | 209.8 | 171.4 | 159.2 | 130.9 |  |
| 40 | 198.0 | 210.0 | 189.8 | 191.2 | 165.5 | 173.3 | 173.8 | 139.8 | 127.7 | 103.2 |  |
| 41 | 139.3 | 147.6 | 133.5 | 134.4 | 116.4 | 121.8 | 122.2 | 98.3 | 89.8 | 72.6 |  |
| 42 | 130.8 | 135.4 | 119.3 | 116.8 | 98.1 | 99.4 | 96.1 | 74.3 | 64.9 | 50.0 |  |
| 43 | 89.9 | 93.4 | 82.6 | 81.2 | 68.5 | 69.7 | 67.9 | 52.8 | 46.5 | 36.1 |  |
| 44 | 83.6 | 85.7 | 74.7 | 72.2 | 59.8 | 59.6 | 56.5 | 42.7 | 36.4 | 27.2 |  |
| 45 | 77.7 | 78.8 | 67.8 | 64.7 | 52.7 | 51.5 | 47.8 | 35.2 | 28.9 | 20.6 |  |
| 46 | 72.7 | 73.6 | 63.2 | 60.1 | 48.8 | 47.6 | 43.9 | 32.1 | 26.2 | 18.5 |  |
| 47 | 78.4 | 78.9 | 67.3 | 63.5 | 51.0 | 49.1 | 44.7 | 32.0 | 25.5 | 17.3 |  |
| 48 | 59.9 | 60.9 | 52.6 | 50.4 | 41.2 | 40.6 | 37.9 | 28.1 | 23.4 | 16.9 |  |
| 49 | 34.1 | 36.1 | 32.5 | 32.7 | 28.3 | 29.6 | 29.6 | 23.8 | 21.6 | 17.5 |  |

***Appendix 5:*** **Numbers of women by age (in thousands) in different years estimated from the data in the 2020 national population census of China**

| Age of women | Year | | | | | | | | | | |
| --- | --- | --- | --- | --- | --- | --- | --- | --- | --- | --- | --- |
|  | 2000 | 2001 | 2002 | 2003 | 2004 | 2005 | 2006 | 2007 | 2008 | 2009 | 2010 |
| 15 | 9596.2 | 10398.0 | 11963.4 | 12270.0 | 12121.3 | 12518.2 | 11225.4 | 9496.2 | 8941.0 | 8381.7 | 8072.6 |
| 16 | 9470.5 | 9591.5 | 10393.2 | 11958.3 | 12265.2 | 12116.8 | 12513.7 | 11221.5 | 9493.0 | 8938.1 | 8379.2 |
| 17 | 10042.8 | 9465.2 | 9586.5 | 10388.2 | 11952.9 | 12259.9 | 12111.9 | 12508.8 | 11217.3 | 9489.6 | 8935.0 |
| 18 | 10063.0 | 10036.6 | 9459.8 | 9581.3 | 10383.0 | 11947.3 | 12254.5 | 12106.7 | 12503.6 | 11212.7 | 9485.9 |
| 19 | 9036.6 | 10056.5 | 10030.4 | 9454.4 | 9576.3 | 10377.8 | 11941.6 | 12248.8 | 12101.3 | 12498.2 | 11208.1 |
| 20 | 9109.4 | 9030.2 | 10049.8 | 10024.2 | 9449.0 | 9571.1 | 10372.5 | 11935.6 | 12242.9 | 12095.7 | 12492.7 |
| 21 | 9384.9 | 9102.5 | 9023.9 | 10043.2 | 10018.0 | 9443.5 | 9565.7 | 10366.9 | 11929.5 | 12236.8 | 12089.9 |
| 22 | 9015.3 | 9377.5 | 9095.7 | 9017.6 | 10036.6 | 10011.8 | 9437.9 | 9560.4 | 10361.3 | 11923.3 | 12230.7 |
| 23 | 9314.5 | 9007.7 | 9370.1 | 9089.0 | 9011.4 | 10030.2 | 10005.7 | 9432.3 | 9554.9 | 10355.6 | 11916.9 |
| 24 | 9968.0 | 9306.3 | 9000.3 | 9362.9 | 9082.5 | 9005.3 | 10023.7 | 9999.4 | 9426.7 | 9549.4 | 10349.9 |
| 25 | 10624.6 | 9959.0 | 9298.4 | 8993.1 | 9355.9 | 9076.1 | 8999.3 | 10017.2 | 9993.2 | 9421.0 | 9543.9 |
| 26 | 11387.0 | 10614.7 | 9950.2 | 9290.7 | 8986.2 | 9349.1 | 9069.8 | 8993.2 | 10010.7 | 9987.0 | 9415.4 |
| 27 | 11819.5 | 11375.9 | 10604.9 | 9941.7 | 9283.2 | 8979.4 | 9342.3 | 9063.5 | 8987.2 | 10004.3 | 9980.8 |
| 28 | 12204.4 | 11807.5 | 11365.1 | 10595.4 | 9933.4 | 9275.9 | 8972.7 | 9335.6 | 9057.1 | 8981.1 | 9997.9 |
| 29 | 12842.5 | 12191.6 | 11795.9 | 11354.6 | 10586.2 | 9925.2 | 9268.6 | 8965.9 | 9328.8 | 9050.7 | 8975.1 |
| 30 | 12868.5 | 12828.5 | 12179.0 | 11784.5 | 11344.2 | 10577.1 | 9917.1 | 9261.3 | 8959.0 | 9321.9 | 9044.4 |
| 31 | 12922.1 | 12853.9 | 12814.8 | 12166.8 | 11773.3 | 11334.1 | 10568.1 | 9908.9 | 9253.9 | 8952.2 | 9315.1 |
| 32 | 12082.5 | 12906.8 | 12839.5 | 12801.2 | 12154.7 | 11762.3 | 11323.9 | 10558.9 | 9900.6 | 9246.5 | 8945.4 |
| 33 | 11453.6 | 12067.6 | 12891.7 | 12825.3 | 12787.9 | 12142.8 | 11751.2 | 11313.6 | 10549.6 | 9892.3 | 9239.1 |
| 34 | 12027.1 | 11438.6 | 12052.7 | 12876.6 | 12811.3 | 12774.6 | 12130.7 | 11739.9 | 11303.2 | 10540.3 | 9883.9 |
| 35 | 11853.8 | 12010.4 | 11423.6 | 12037.8 | 12861.5 | 12797.1 | 12761.0 | 12118.3 | 11728.4 | 11292.5 | 10530.8 |
| 36 | 12664.5 | 11836.5 | 11993.8 | 11408.6 | 12023.0 | 12846.5 | 12782.7 | 12747.1 | 12105.6 | 11716.6 | 11281.7 |
| 37 | 11822.1 | 12644.8 | 11819.0 | 11977.0 | 11393.5 | 12008.0 | 12831.0 | 12767.9 | 12732.9 | 12092.7 | 11704.7 |
| 38 | 7896.6 | 11802.6 | 12624.9 | 11801.4 | 11960.1 | 11378.2 | 11992.5 | 12815.0 | 12752.7 | 12718.4 | 12079.4 |
| 39 | 6292.8 | 7882.8 | 11782.8 | 12604.6 | 11783.5 | 11942.9 | 11362.5 | 11976.6 | 12798.7 | 12737.1 | 12703.5 |
| 40 | 6646.1 | 6280.9 | 7868.7 | 11762.8 | 12584.2 | 11765.4 | 11925.3 | 11346.4 | 11960.2 | 12781.8 | 12721.1 |
| 41 | 7322.9 | 6632.8 | 6268.9 | 7854.4 | 11742.5 | 12563.4 | 11746.7 | 11907.0 | 11329.6 | 11943.4 | 12764.6 |
| 42 | 8759.8 | 7307.0 | 6619.0 | 6256.5 | 7839.8 | 11721.5 | 12541.7 | 11727.2 | 11888.0 | 11312.2 | 11925.9 |
| 43 | 8957.5 | 8739.2 | 7290.5 | 6604.9 | 6243.7 | 7824.6 | 11699.6 | 12519.0 | 11706.8 | 11868.2 | 11294.1 |
| 44 | 8839.3 | 8934.7 | 8717.8 | 7273.4 | 6590.1 | 6230.3 | 7808.6 | 11676.5 | 12495.2 | 11685.4 | 11847.4 |
| 45 | 9126.7 | 8814.6 | 8910.8 | 8695.4 | 7255.5 | 6574.6 | 6216.1 | 7791.6 | 11652.0 | 12470.0 | 11662.9 |
| 46 | 8819.0 | 9098.9 | 8788.8 | 8885.8 | 8671.9 | 7236.7 | 6558.2 | 6201.2 | 7773.7 | 11626.3 | 12443.4 |
| 47 | 8471.5 | 8789.7 | 9069.8 | 8761.8 | 8859.6 | 8647.4 | 7216.9 | 6541.0 | 6185.4 | 7754.9 | 11599.2 |
| 48 | 7921.5 | 8441.0 | 8759.2 | 9039.4 | 8733.5 | 8832.1 | 8621.5 | 7196.1 | 6522.9 | 6169.0 | 7735.3 |
| 49 | 7172.0 | 7890.5 | 8409.0 | 8727.3 | 9007.7 | 8704.1 | 8803.5 | 8594.4 | 7174.3 | 6504.0 | 6151.8 |
| Age of women | Year | | | | | | | | | | |
|  | 2011 | 2012 | 2013 | 2014 | 2015 | 2016 | 2017 | 2018 | 2019 | 2020 |  |
| 15 | 7829.4 | 7383.7 | 7213.4 | 6871.7 | 6693.2 | 6767.4 | 6596.4 | 6409.9 | 6678.0 | 7956.2 |  |
| 16 | 8070.3 | 7827.3 | 7381.8 | 7211.5 | 6870.0 | 6691.6 | 6765.9 | 6595.0 | 6408.6 | 7562.1 |  |
| 17 | 8376.4 | 8067.7 | 7824.9 | 7379.6 | 7209.6 | 6868.2 | 6689.9 | 6764.2 | 6593.4 | 7063.3 |  |
| 18 | 8931.8 | 8373.5 | 8065.0 | 7822.4 | 7377.4 | 7207.5 | 6866.3 | 6688.2 | 6762.5 | 6608.8 |  |
| 19 | 9482.2 | 8928.5 | 8370.4 | 8062.2 | 7819.8 | 7375.1 | 7205.3 | 6864.4 | 6686.3 | 7283.3 |  |
| 20 | 11203.4 | 9478.4 | 8925.0 | 8367.4 | 8059.4 | 7817.2 | 7372.7 | 7203.1 | 6862.2 | 7121.3 |  |
| 21 | 12487.1 | 11198.5 | 9474.5 | 8921.4 | 8364.2 | 8056.5 | 7814.5 | 7370.3 | 7200.7 | 6864.4 |  |
| 22 | 12084.1 | 12481.3 | 11193.6 | 9470.5 | 8917.9 | 8361.0 | 8053.6 | 7811.7 | 7367.7 | 6779.1 |  |
| 23 | 12224.5 | 12078.3 | 12475.5 | 11188.6 | 9466.5 | 8914.3 | 8357.8 | 8050.6 | 7808.9 | 7077.4 |  |
| 24 | 11910.7 | 12218.3 | 12072.5 | 12469.8 | 11183.6 | 9462.5 | 8910.7 | 8354.5 | 8047.5 | 7624.0 |  |
| 25 | 10344.2 | 11904.4 | 12212.1 | 12066.7 | 12464.1 | 11178.8 | 9458.6 | 8907.1 | 8351.2 | 7712.6 |  |
| 26 | 9538.5 | 10338.6 | 11898.2 | 12206.1 | 12061.0 | 12458.5 | 11173.9 | 9454.6 | 8903.5 | 8009.7 |  |
| 27 | 9409.8 | 9533.0 | 10333.0 | 11892.1 | 12200.1 | 12055.4 | 12452.9 | 11169.1 | 9450.6 | 8734.5 |  |
| 28 | 9974.6 | 9404.3 | 9527.7 | 10327.5 | 11886.1 | 12194.2 | 12049.7 | 12447.3 | 11164.1 | 9341.9 |  |
| 29 | 9991.4 | 9968.5 | 9398.8 | 9522.4 | 10322.0 | 11880.0 | 12188.2 | 12044.1 | 12441.6 | 11104.7 |  |
| 30 | 8969.1 | 9985.1 | 9962.4 | 9393.3 | 9517.1 | 10316.6 | 11874.0 | 12182.2 | 12038.3 | 12695.9 |  |
| 31 | 9038.1 | 8963.1 | 9978.7 | 9956.4 | 9387.9 | 9511.9 | 10311.1 | 11868.0 | 12176.2 | 12048.1 |  |
| 32 | 9308.3 | 9031.8 | 8957.2 | 9972.4 | 9950.3 | 9382.5 | 9506.7 | 10305.7 | 11861.9 | 12461.5 |  |
| 33 | 8938.5 | 9301.5 | 9025.4 | 8951.2 | 9966.1 | 9944.3 | 9377.1 | 9501.3 | 10300.1 | 11769.3 |  |
| 34 | 9231.5 | 8931.5 | 9294.6 | 9019.0 | 8945.2 | 9959.7 | 9938.2 | 9371.5 | 9495.9 | 10280.7 |  |
| 35 | 9875.4 | 9223.9 | 8924.6 | 9287.7 | 9012.7 | 8939.2 | 9953.3 | 9932.0 | 9365.8 | 9473.1 |  |
| 36 | 10521.1 | 9866.7 | 9216.2 | 8917.5 | 9280.6 | 9006.1 | 8933.0 | 9946.7 | 9925.6 | 9235.2 |  |
| 37 | 11270.6 | 10511.3 | 9858.0 | 9208.4 | 8910.3 | 9273.5 | 8999.5 | 8926.7 | 9939.8 | 9798.6 |  |
| 38 | 11692.4 | 11259.4 | 10501.3 | 9849.0 | 9200.5 | 8903.0 | 9266.2 | 8992.7 | 8920.1 | 10290.5 |  |
| 39 | 12065.9 | 11679.8 | 11247.8 | 10491.0 | 9840.0 | 9192.4 | 8895.5 | 9258.7 | 8985.6 | 8486.1 |  |
| 40 | 12688.2 | 12052.1 | 11667.1 | 11236.2 | 10480.7 | 9830.7 | 9184.1 | 8887.8 | 9250.8 | 9187.9 |  |
| 41 | 12704.6 | 12672.5 | 12037.8 | 11654.0 | 11224.3 | 10470.1 | 9821.1 | 9175.5 | 8879.7 | 9033.7 |  |
| 42 | 12746.6 | 12687.7 | 12656.3 | 12023.2 | 11640.6 | 11211.9 | 10459.0 | 9811.1 | 9166.4 | 8702.9 |  |
| 43 | 11907.8 | 12728.1 | 12670.0 | 12639.6 | 12008.1 | 11626.7 | 11199.0 | 10447.4 | 9800.6 | 9037.3 |  |
| 44 | 11275.2 | 11888.8 | 12708.6 | 12651.6 | 12622.1 | 11992.3 | 11612.0 | 11185.4 | 10435.0 | 9632.1 |  |
| 45 | 11825.6 | 11255.3 | 11868.8 | 12688.3 | 12632.5 | 12603.9 | 11975.7 | 11596.4 | 11170.7 | 10165.6 |  |
| 46 | 11639.2 | 11802.6 | 11234.4 | 11847.9 | 12667.0 | 12612.3 | 12584.5 | 11957.9 | 11579.6 | 10977.7 |  |
| 47 | 12415.6 | 11614.3 | 11778.5 | 11212.5 | 11826.1 | 12644.7 | 12591.0 | 12563.9 | 11938.9 | 11550.6 |  |
| 48 | 11571.0 | 12386.6 | 11588.5 | 11753.5 | 11190.0 | 11803.5 | 12621.3 | 12568.5 | 12542.1 | 11638.2 |  |
| 49 | 7714.8 | 11541.7 | 12356.5 | 11561.8 | 11727.9 | 11166.6 | 11779.9 | 12596.8 | 12544.8 | 12246.0 |  |

***Appendix 6:* Trends in the estimated and reported total fertility rates (TFRs) and the estimated number of annual births from 2000 to 2020 in China**

| **Year** | **Reported TFRs** | **Estimated TFRs** | **Estimated number of annual births**  **(in millions)** |
| --- | --- | --- | --- |
| 2000 | 1.22 | 1.43 | 15.07 |
| 2001 | 1.39 | 1.44 | 14.84 |
| 2002 | 1.39 | 1.43 | 14.48 |
| 2003 | 1.41 | 1.42 | 14.14 |
| 2004 | 1.45 | 1.58 | 15.65 |
| 2005 | 1.34 | 1.60 | 15.73 |
| 2006 | 1.38 | 1.68 | 16.59 |
| 2007 | 1.45 | 1.72 | 17.08 |
| 2008 | 1.48 | 1.77 | 17.76 |
| 2009 | 1.37 | 1.77 | 18.09 |
| 2010 | 1.19 | 1.71 | 17.71 |
| 2011 | 1.03 | 1.74 | 18.14 |
| 2012 | 1.25 | 1.89 | 19.72 |
| 2013 | 1.22 | 1.77 | 18.28 |
| 2014 | 1.26 | 1.84 | 18.90 |
| 2015 | 1.05 | 1.66 | 16.81 |
| 2016 | 1.24 | 1.80 | 18.09 |
| 2017 | 1.58 | 1.88 | 18.67 |
| 2018 | 1.50 | 1.58 | 15.46 |
| 2019 | 1.47 | 1.51 | 14.55 |
| 2020 | 1.30 | 1.30 | 12.13 |

***Appendix 7:*** **Trends and differences in the reported and estimated total fertility rates (TFRs) between rural and urban areas from 2000 to 2020 in China**

| **Year** | **Reported rural TFRs** | **Reported urban TFRs** | **Estimated rural TFRs** | **Estimated urban TFRs** | **Rural-urban difference** |
| --- | --- | --- | --- | --- | --- |
| 2000 | 1.43 | 0.94 | 1.68 | 1.09 | 0.58 |
| 2001 | 1.60 | 1.07 | 1.65 | 1.14 | 0.51 |
| 2002 | 1.65 | 1.03 | 1.69 | 1.09 | 0.61 |
| 2003 | 1.68 | 1.08 | 1.68 | 1.10 | 0.58 |
| 2004 | 1.69 | 1.15 | 1.83 | 1.27 | 0.56 |
| 2005 | 1.65 | 1.04 | 1.97 | 1.25 | 0.73 |
| 2006 | 1.72 | 1.06 | 2.08 | 1.29 | 0.79 |
| 2007 | 1.75 | 1.17 | 2.07 | 1.39 | 0.68 |
| 2008 | 1.74 | 1.23 | 2.07 | 1.49 | 0.58 |
| 2009 | 1.61 | 1.15 | 2.06 | 1.50 | 0.56 |
| 2010 | 1.44 | 0.98 | 2.10 | 1.40 | 0.70 |
| 2011 | 1.34 | 0.81 | 2.25 | 1.36 | 0.88 |
| 2012 | 1.58 | 1.01 | 2.41 | 1.52 | 0.89 |
| 2013 | 1.57 | 0.98 | 2.29 | 1.42 | 0.87 |
| 2014 | 1.60 | 1.03 | 2.38 | 1.50 | 0.88 |
| 2015 | 1.27 | 0.91 | 2.02 | 1.45 | 0.57 |
| 2016 | 1.43 | 1.13 | 2.08 | 1.64 | 0.44 |
| 2017 | 1.74 | 1.49 | 2.06 | 1.77 | 0.29 |
| 2018 | 1.69 | 1.38 | 1.78 | 1.46 | 0.32 |
| 2019 | 1.71 | 1.33 | 1.75 | 1.37 | 0.38 |
| 2020 | 1.54 | 1.20 | 1.54 | 1.20 | 0.35 |

***Appendix 8:*** **Comparison of estimated total fertility rates (TFRs) between 2000 and 2020, stratified by region and urban-rural areas**

| **Region** | **Total** | | **Rural areas** | | **Urban areas** | |
| --- | --- | --- | --- | --- | --- | --- |
|  | **2010** | **2020** | **2010** | **2020** | **2010** | **2020** |
| North | 1.63 | 1.17 | 2.06 | 1.32 | 1.31 | 1.11 |
| Northeast | 1.07 | 0.86 | 1.29 | 0.80 | 0.92 | 0.87 |
| East | 1.67 | 1.23 | 2.05 | 1.44 | 1.42 | 1.16 |
| Central | 1.91 | 1.33 | 2.19 | 1.54 | 1.59 | 1.22 |
| South | 1.76 | 1.49 | 2.37 | 2.04 | 1.45 | 1.33 |
| Southwest | 1.88 | 1.48 | 2.21 | 1.70 | 1.49 | 1.35 |
| Northwest | 1.88 | 1.32 | 2.26 | 1.44 | 1.42 | 1.25 |

***Appendix 9:* Trends in the estimated total fertility rates (TFRs) by robust check**

| **Year** | **Estimated TFRs** | **Estimated rural TFRs** | **Estimated urban TFRs** |
| --- | --- | --- | --- |
| 2000 | 1.43 | 1.68 | 1.09 |
| 2001 | 1.44 | 1.64 | 1.14 |
| 2002 | 1.43 | 1.68 | 1.09 |
| 2003 | 1.41 | 1.66 | 1.10 |
| 2004 | 1.57 | 1.80 | 1.27 |
| 2005 | 1.58 | 1.94 | 1.24 |
| 2006 | 1.67 | 2.06 | 1.28 |
| 2007 | 1.71 | 2.06 | 1.38 |
| 2008 | 1.77 | 2.06 | 1.48 |
| 2009 | 1.77 | 2.06 | 1.50 |
| 2010 | 1.71 | 2.10 | 1.40 |
| 2011 | 1.74 | 2.24 | 1.36 |
| 2012 | 1.89 | 2.40 | 1.52 |
| 2013 | 1.76 | 2.27 | 1.41 |
| 2014 | 1.82 | 2.34 | 1.49 |
| 2015 | 1.63 | 1.98 | 1.42 |
| 2016 | 1.79 | 2.05 | 1.62 |
| 2017 | 1.88 | 2.05 | 1.77 |
| 2018 | 1.58 | 1.78 | 1.46 |
| 2019 | 1.51 | 1.76 | 1.38 |
| 2020 | 1.30 | 1.54 | 1.20 |
